# Provider perspectives on demand creation for maternal vaccines in Kenya

**DOI:** 10.12688/gatesopenres.12833.1

**Published:** 2018-07-19

**Authors:** Irina Bergenfeld, Stacy W. Nganga, Courtni A. Andrews, Vincent L. Fenimore, Nancy A. Otieno, Andrew D. Wilson, Sandra S. Chaves, Jennifer R. Verani, Marc-Alain Widdowson, Winnie N. Wairimu, Susan N. Wandera, Raphael O. Atito, Maxwell O. Adero, Paula M. Frew, Saad B. Omer, Fauzia A. Malik

**Affiliations:** 1 Hubert Department of Global Health, Emory University, Atlanta, GA, 30322, USA; 2 Division of Global Health, Kenya Medical Research Institute, Kisumu, Kenya; 3 Division of Global Health Protection, Centers for Disease Control and Prevention, Kenya Office, Nairobi, Kenya; 4 National Center for Immunization and Respiratory Diseases, Centers for Disease Control and Prevention, Kenya Office, Nairobi, Kenya; 5 Division of Infectious Diseases, Emory University, Atlanta, GA, 30322, USA; 6 Department of Behavioral Science and Health Education, Emory University, Atlanta, GA, 30322, USA; 7 Department of Epidemiology, Emory University, Atlanta, GA, 30322, USA; 8 Department of Pediatrics, Emory University, Atlanta, GA, 30322, USA

**Keywords:** Maternal Immunization, Maternal and Child Health, Health Care Providers, Kenya, Developing Countries

## Abstract

**Background** *. *Expansion of maternal immunization, which offers some of the most effective protection against morbidity and mortality in pregnant women and neonates, requires broad acceptance by healthcare providers and their patients. We aimed to describe issues surrounding acceptance and demand creation for maternal vaccines in Kenya from a provider perspective.

**Methods** *.* Nurses and clinical officers were recruited for semi-structured interviews covering resources for vaccine delivery, patient education, knowledge and attitudes surrounding maternal vaccines, and opportunities for demand creation for new vaccines. Interviews were conducted in English and Swahili, transcribed verbatim from audio recordings, and analyzed using codes developed from interview guide questions and emergent themes.

**Results** *.* Providers expressed favorable attitudes about currently available maternal immunizations and introduction of additional vaccines, viewing themselves as primarily responsible for vaccine promotion and patient education.  The importance of educational resources for both patients and providers to maintain high levels of maternal immunization coverage was a common theme. Most identified barriers to vaccine acceptance and delivery were cultural and systematic in nature. Suggestions for improvement included improved patient and provider education, including material resources, and community engagement through religious and cultural leaders.

**Conclusions** *.* The distribution of standardized, evidence-based print materials for patient education may reduce provider overwork and facilitate in-clinic efforts to inform women about maternal vaccines. Continuing education for providers should address communication surrounding current vaccines and those under consideration for introduction into routine schedules. Engagement of religious and community leaders, as well as male decision-makers in the household, will enhance future acceptance of maternal vaccines.

## Introduction

Reductions in neonatal mortality have not kept pace with overall declines in childhood mortality, partly because neonates are too young to be vaccinated, and have therefore not benefitted from the progressive introduction of childhood vaccines globally ^1^. Maternal immunizations offer some of the most effective protection against morbidity and mortality in both pregnant women and young infants ^2^. The promise to confer additional protection to young infants via maternal immunization is bolstered as new vaccine candidates, such as respiratory syncytial virus (RSV), are being identified; field and clinic strategies are being employed to increase vaccine access; and behavioral-communication research findings are implemented to improve vaccine messaging strategies ^2^. Expansion of maternal immunization coverage, however, requires broad acceptance by both pregnant women and their healthcare providers (HCPs) ^3, 4^.

Kenya continues to contend with challenges that threaten previous gains made in immunization coverage, with noted drops in recent coverage of childhood vaccines since 2011 ^5^. One effective approach to maintain high coverage rates involves enabling providers to use effective messaging during patient interactions. Such a strategy builds upon patient knowledge, aids in countering misinformation, and provides a trusted source for women to confide in on maternal and child health visits ^6^. Although quantitative surveys, and, to a lesser degree, qualitative studies, have contributed knowledge to address current issues regarding maternal immunization uptake in high-income settings ^7^, there is a dearth of research considering low- and middle-income countries.

Parents consistently rank healthcare providers as their most trusted source of vaccine information for both maternal and childhood vaccines ^8– 11^. The centrality of the patient-provider relationship in the promotion of immunization necessitates a comprehensive and nuanced understanding of how this relationship influences both acceptance and demand among patients. The purpose of this study is to describe issues surrounding vaccine acceptance and demand creation from a provider perspective, to inform future efforts to introduce new maternal vaccines in low- and middle-income countries. Our analysis draws on data from a large, in-depth study of determinants of maternal vaccine acceptance in Kenya.

## Results

Of the final sample of 111 HCPs, 37.5% were currently working at public facilities. All HCPs interviewed were nurses (n=97) or clinical officers (n=14). Six major themes emerged from interviews: (1) the centrality of the patient-provider relationship in vaccine promotion; (2) cultural, religious, and social factors influencing vaccine acceptance among patients; (3) resources needed for improved vaccine delivery; (4) differences in provider knowledge about maternal vaccines; (5) favorable attitudes toward maternal vaccines; (6) patient access issues. Note that quotes presented in this manuscript and accompanying tables have been copyedited to improve readability. All efforts have been made to ensure the original meaning has not been changed. Direct quotes are available in the original transcripts ( Dataset 1).

### The centrality of the patient-provider relationship

Providers expressed that they were pregnant women’s primary source of health information, and that it was therefore their duty to educate pregnant women about maternal vaccines ( Table 1). Many providers expressed confidence that patients would accept their instructions without question, especially in rural facilities where patients are felt to have more limited knowledge. One provider in Marsabit expresses this view:

*“I guess some people don’t have the understanding of the vaccines. So when they come here, they cite the myths and misconceptions about vaccines that they have been fed back in their villages.”*

Providers also admitted that the relative social position of healthcare providers and patients could be a factor in high acceptance, as pregnant women are expected to defer to provider’s greater knowledge and authority in health matters. In the words of one Nairobi provider:

*“Maybe sometimes they do not have that chance to say no because they look at me as their savior… and everything I tell them, they believe is right.”*

However, some acknowledged that expectations of total patient deference were changing as women become more comfortable initiating requests for information. This openness to two-way exchange was more commonly expressed among urban providers, such as this Nairobi-based nurse:

*“In the past, people used to be blasted by the nurses or whoever was giving the services whenever they asked questions. Those days are long gone. It is always good to ask why you are being injected.”*

**Table 1.** Centrality of patient-provider relationship in vaccine promotion.

*Subtheme*	*Quote(s)*
*Provider Duty*	*We advise mothers because there is a lot they do not know when they come here. It is our responsibility to* *advise them; we tell them ‘this is what you are supposed to do.’*
*Perceptions of implicit* *patient trust*	*You realize that TT (tetanus toxoid) uptake is increased. [Pregnant women] are confident with what we tell them* *and we are also confident that their attitude is positive. This is evident in the fact that they come in numbers for* *the vaccines. In some cases, they come from other hospitals. They trust us.* *The pregnant mothers always trust [healthcare personnel] because anything they are told to do concerning their* *health they will do.*
*Expectations of patient* *deference*	*For most mothers they, just do what you tell them to do, like if they are not aware that these vaccines are* *supposed to be given, they will not ask for it… It is the healthcare worker who is supposed to sensitize them that* *there is a vaccine like this one and it does such and such a thing and it is good for you, and mostly they do not* *say no to it.*
*Evolving patient/* *provider roles*	*As you know, these are mature people and you cannot just inject them, you first talk to them and give them the* *injection if they accept.*

### Cultural, religious, and social factors influencing vaccine acceptance

Some providers highlighted the limited agency of pregnant women regarding their own medical decision-making. For example, a woman might be unable to access types of care that run contrary to her husband’s beliefs ( Table 2). Conversely, husbands and male relatives who were involved in women’s antenatal care could be facilitators of maternal vaccination.

Providers cited community and religious institutions as both barriers and facilitators of vaccine acceptance among their patients. Religious institutions could provide welcome venues for vaccine education outreach, while traditional leadership could serve as gatekeepers to community acceptance. One provider in Marsabit suggested that community meetings would be an ideal venue to reach those who might otherwise not come for antenatal classes (ANC):

“ *The other thing that can be done is to communicate the importance of vaccines through the chiefs during the **barazas** [community meetings] where even the county officials can explain it to them.”*

Other providers identified certain religious denominations as barriers to vaccine acceptance, stating that the leaders of these groups instructed followers to avoid medical treatment generally or vaccines specifically. One Nairobi provider cites a traditional religious denomination as one such group:

*“During the polio campaigns, the Akorino rejected it in Eastern Kenya because their religion does not allow them to take medication.”*

**Table 2.** Cultural, religious, and social factors influencing vaccine acceptance.

*Subtheme*	*Quote(s)*
*Opportunities for health* *education*	*Where I come from, there are usually sessions every Sunday in our church where there is a healthcare* *provider who talks to the church about issues that arise in the health sector. I think that would work so well* *because you would capture so many mothers on a Sunday as compared to when you expect them to come to* *clinic every day.*
*Influence of religious and* *community leaders*	*Cervical cancer [vaccine] was launched last year for girls from nine years old, and a Catholic pope [sic] said* *it is linked to family planning. It happens among a few churches or religions, but once it is clarified, a few* *receive [it], while others refuse based on what their spiritual fathers are telling them. Generally, whatever is* *approved and announced important by the government is accepted.* *Let me say that every community has its own culture and taboos. This can be addressed [in] the community* *by first sitting down with the leaders and talking to them about it. If you find a better way of talking to the* *community leaders, then you have a better way [of reaching] the community, because the community listen to* *their leaders[more] than the outsiders because they believe in them.*
*Limitations on women’s* *agency*	***She got married to guy who prevented her from going for [antenatal] clinic.** I asked her why she* *didn’t want to go for ANC services for her first born! I urged her that it was important to get vaccinated. She* *told me that their church doctrine restrains them from going to hospital. They get such instructions from* *their pastors. **I called in her brothers and explained to them the importance of tetanus vaccine from a*** ***medical perspective.** I also discussed with them antenatal care and the need for health education. **After that*** ***engagement with family members, they forcefully took her to hospital.***

### Resources needed for improved vaccine delivery

Providers almost universally cited time constraints and provider overwork as barriers to providing adequate patient education about vaccines ( Table 3), with many suggesting that human resource allocation was the major limitation faced by their clinics in vaccine delivery. Many complained of overwork and long lines, factors seen as contributing to patient attrition from their clinics. In the words of a nurse at a private facility:

*“We have had couples leaving because of the high queues.”*

Educational materials for patients were often cited as absent or inadequate, a situation that was often blamed on the shifting responsibilities to county government following devolution (political decentralization) of powers from national to county governance in 2010 ( Table 3). Some providers suggested that patient brochures needed to be translated into local languages, as women at their clinics did not read English or Swahili. One provider in rural Marsabit explains:

“ *I think the information should be printed out in different languages and given to the community radio stations so that it can be explained to them, because they do not understand Swahili.”*

Likewise, providers expressed a desire for continuing professional education, particularly on newer vaccines. One provider links professional education to patient acceptance:

*“If you do not update me properly, it will be of no use even if [a new vaccine] is brought to the facility. It will be available but I will not give it out, not because the patients are not asking for it, but because it is me who is not interested in giving it out.”*

Some providers also raised concerns about shortages of physical resources, also linking these to ongoing decentralization efforts as counties accepted greater financial responsibility for vaccine delivery and administration.

**Table 3.** Resources needed for improved vaccine delivery.

*Subtheme*	*Quote(s)*
*Human resources*	*Maybe when a health care provider is in a hurry or is being overworked, you may find a long queue at the ANC* *waiting for vaccination. The nurse there may not have time to discuss much with every client about the vaccines.* *Sometimes they issue orders for the mothers to queue and get vaccinated. These are situations which may* *happen when there are several mothers at the clinic. This can cripple vaccine uptake since there is no time for* *explanations.*
*Continuing provider* *education*	*…healthcare workers should be taken for regular updates on immunization. Most of us study on [the] job. There is* *probability of missing some items during on-the-job training. There are those gaps, and if the health care provider* *is updated, those gaps will be removed.*
*Patient Education*	*Before the Division of Vaccines and Immunization, Nairobi used to give us educational materials, handbills for the* *mothers. Since **devolution** [political decentralization] we have not had anything to give to the mothers to carry* *home.*
*Material resources* *post-decentralization*	*Human resources, then the cold chain equipment like the vaccine carrier the cold boxes. We need the* *refrigerators and syringes and other logistics. We need money. Actually finance is the first and foremost…* *Currently with devolution, it means the county has to contribute, so at any given time if they do not contribute* *and leave it to the national level then we might even fail. So I think we might get challenges in the long run with* *devolution, but we hope not.*

### Differences in provider knowledge about maternal immunizations

While the overwhelming majority of providers are comfortable in their knowledge of vaccines currently in the national schedule and available at their facilities, few are knowledgeable about vaccines not offered at their facilities, or about upcoming vaccines ( Table 4). For example, providers at private facilities, which are likely to carry influenza vaccines, were more familiar with this vaccine than those at public facilities, where influenza vaccine is generally not available:

*“It [flu vaccine] is mostly given by the private facilities. They say it is okay. I will, however, not vote for it or say much about it because I have never issued it.”*

**Table 4.** Differences in provider knowledge about maternal immunizations.

*Subtheme*	*Quote(s)*
*Confidence in knowledge of* *current vaccines*	*If you go a long time without being updated on current trends or emerging issues in immunization,* *you may not have the right confidence, but for now, I think I am well suited to handle anything.*
*Lack of familiarity with* *vaccines not currently in the* *schedule*	*Is there any other vaccine apart from tetanus? I have not heard of any other.*

### Favorable attitudes toward maternal vaccines

The vast majority of providers expressed the view that tetanus toxoid was beneficial to pregnant women and infants, and were open to new vaccines being introduced ( Table 5). They also believed that most healthcare providers shared their positive attitudes. Providers were less comfortable endorsing vaccines with which they were not personally familiar, especially those at public facilities. A few stated that they already received complaints from pregnant women regarding both TT2+ and childhood vaccines and that introducing more vaccines would exacerbate this problem. One Mombasa-based provider expresses doubt that pregnant women will readily accept new vaccines:

*“If they are already complaining that the available vaccines are too much or painful, how about additional vaccines? That is the concern. There will be more complaints regarding additional vaccines.”*

**Table 5.** Favorable attitudes toward maternal vaccines.

Subtheme	Quote(s)
*Perceptions of positive* *attitudes among providers*	*I would say [maternal vaccines] are effective, and if mothers got information about the importance of early* *childhood immunizations, I think we would go far… Most of them [healthcare providers] will talk positively* *of vaccines.*
*Openness to new vaccines*	*Pregnant women are prone to many diseases because their immunity is low. If they could be given a* *vaccine that could boost their immunity, it would be better, because they are prone to UTI (urinary tract* *infection).*
*Attitudes linked to familiarity*	*[Flu vaccine] is mostly given by the private facilities. They say it is okay. I will, however, not vote for it or say* *much about it because I have never issued it.*

### Patient access issues

Distance to health facilities and lack of transportation was one of the major access issues perceived by providers to hinder maternal vaccine coverage ( Table 6). Other providers cited the cost of medical services at private facilities and inter-clinic mobility as sources of patient attrition during antenatal care. In the words of one Mombasa-based provider,

*“Private hospitals are established to make profits. The charges may keep them away.”*

Urban providers tended to express more frustration with patient mobility, as women in these settings often have access to several facilities offering the same services. This was linked to the notion that women often move during their pregnancy, leaving gaps in their medical records that are difficult to address without an integrated system. One Nairobi provider describes how patient mobility impacts coverage of TT2+ booster doses:

“ *The only challenge is that the subsequent doses like when the women delivers, coming for the other doses is a challenge and you know this is cosmopolitan town so people come… Somebody would deliver and they would even go away to their homes maybe in another county so we miss them out for the subsequent doses.”*

**Table 6.** Patient access issues.

*Subtheme*	*Quote(s)*
*Distance to health* *facility*	*Some do not come to the clinic may be because of the distance. For instance, in the rural areas you would find a* *mother delivering at home assisted by a TBA [traditional birth attendant] and not going to the facility, maybe just* *because of the distance or financial issues, which can also contribute.*
*Cost*	*We have high dropouts due to financial implications and they go to the public institutions.*
*Patient mobility and* *record-keeping*	*I wish mothers could have cards to actually indicate that they have been vaccinated. It would be easier if we went* *paperless and had a database where you could click to verify one’s vaccination history instead of trusting their* *word. There are some people who have the five doses of tetanus even before delivery and you will have no way of* *verifying it.* *Tracing those who have defaulted is very difficult because people move up and down, and you are not sure if they* *get the same advice and consistency of the shots where they went.*

## Discussion

Healthcare providers expressed favorable attitudes about currently available maternal immunizations, and the majority were open to providing new vaccines that may be in the pipeline, e.g. RSV. In addition, providers viewed themselves as primarily responsible for promoting vaccine acceptance and providing vaccine information to their patients. Nurses and clinical officers alike highlighted the importance of educational resources for both patients and providers to maintain high levels of maternal immunization coverage. Most of the barriers to vaccine acceptance and delivery identified by HCPs were cultural and systematic in nature, including religious objections, time constraints, and financial challenges.

The effectiveness of detailed print materials describing the benefits and risks of childhood vaccinations has been demonstrated in randomized trials ^12^. Providers in our sample emphasized the need for educational resources to facilitate in-clinic interactions with pregnant women, which are often limited by time constraints. This could mitigate some provider overwork issues by giving patients evidence-based materials that could be taken home. Although the Kenyan government has published a maternal and child health booklet that includes some vaccine information, supply issues have limited patient access. These shortages have been exacerbated following political decentralization as counties take on roles previously allocated to the national government. Future efforts to publish educational materials at county level could more easily be tailored to local needs. Providers suggested that multimedia and local language options for patient education materials would improve accessibility and impact, especially among women with limited English and Swahili literacy.

Continuing professional education to gain familiarity with vaccines other than tetanus toxoid was a major need cited by providers. Attitudes towards familiar vaccines are already overwhelmingly positive among providers, but some expressed hesitancy about vaccines that they had no experience administering. Studies have shown that when providers are confident in their own knowledge of medical services such as vaccines, they are more likely to effectively promote these services to their patients ^11, 13^. Such professional updates need to be proactive so that providers are educated ahead of public vaccination campaigns and are prepared to deal with patient questions and concerns. This will be especially critical if new and less familiar vaccines, such as RSV and influenza, are introduced into national immunization schedules in the near future. It may also be beneficial to incorporate communication strategies into ongoing professional education efforts to empower providers to use their limited time with patients more effectively.

Finally, providers highlighted the need to engage religious and community leaders as allies in demand creation and promotion of new vaccines. As the drastic decline in polio vaccine coverage in northern Nigeria demonstrates, failure to elicit buy-in from influential religious groups can result in large-scale failure to promote even already familiar vaccines ^14^. Similarly, recent efforts to introduce HPV vaccination in Kenya have met with resistance from Catholic leadership ( Table 2). Providers suggested that religious groups could be engaged as allies to reach women who might otherwise not receive health messaging. Future efforts to support maternal vaccination should also include male decision-makers, including husbands, fathers, and brothers.

### Limitations

Because a convenience sample of providers was interviewed, it is possible that selection bias towards those with higher vaccine knowledge or more positive vaccine attitudes occurred. Moreover, many providers were recruited and interviewed from their homes due to an ongoing healthcare workers’ strike during the data collection period. Among public sector workers, the strike may also have influenced some responses, particularly those pertaining to working conditions. Private facility workers may also have been experiencing overwork and higher patient volumes due to closures of public facilities. In addition, more HCPs from private facilities not participating in the strike were recruited for interviews, resulting in oversampling from private facilities.

### Conclusions

To maintain high coverage of TT2+ and to ensure widespread acceptance of future maternal vaccines, providers must be empowered to use effective, pro-vaccine messaging in their interactions with pregnant women. Communication strategies for vaccine promotion can be incorporated into current professional education efforts. Print materials for patient education may also facilitate these interactions, and political decentralization provides opportunities and challenges for county-level government to publish regionally tailored resources for patients and providers. Finally, cultural and religious influencers should be leveraged to facilitate the introduction of new maternal vaccines.

## Methods

### Study design

This analysis draws from a large, multi-component, mixed methods study of determinants of maternal vaccine acceptance, which includes data from pregnant women, healthcare providers, and key informants in Kenya. This paper considers only qualitative data from healthcare providers; however, triangulation with pregnant woman and key informant data was used to validate results.

Semi-structured interviews were conducted at 15 facilities in four diverse geographic areas. An interview guide ( Supplementary File 1) was developed using a grounded theory approach drawing on observational data gathered in antenatal clinics (ANCs), literature review, and themes emerging from interviews of pregnant women. This approach was chosen to limit investigator bias and to account for a lack of qualitative vaccine acceptance literature relevant to the Kenyan context. Interviews covered topics including resources for vaccine delivery, experiences with patient education, knowledge of vaccines, and strategies for demand creation. This study was approved by the Institutional Review Boards at Emory University [IRB00089673], the Centers for Disease Control and Prevention (CDC) [covered under reliance agreement with Emory], and the Kenya Medical Research Institute (KEMRI).

### Setting and population

Kenya was chosen as the site of the in-depth phase of the study due to its large birth cohort, relatively high vaccine coverage, and longstanding collaboration with the CDC through KEMRI. Interviews were conducted in four geographically diverse locations in country: Marsabit, Nairobi, Mombasa, and Siaya. These sites were purposively selected to create a sample representing a range of urban and rural locations ( Table 7). A convenience sample of healthcare providers representing 37 public and private facilities was recruited at each site until data saturation was reached; all providers who consented to participate were interviewed. Inclusion criteria were current employment as a healthcare provider, proficiency in English or Swahili, and ability to grant informed consent. Healthcare providers were defined as physicians, nurses, community health workers, and clinical officers (non-physician clinicians licensed by the Kenyan government who undergo additional training beyond that of nurses ^15^).

**Table 7.** Description of facilities and interviews conducted by study site.

Region	Rural/Urban	Public Facilities	Private Facilities	Total Facilities	Nurses	Clinical Officers	Total Interviews
**Nairobi**	Urban	1	10	11	28	4	32
**Siaya**	Rural	5	7	12	21	7	28
**Marsabit**	Rural	1	4	5	12	3	15
**Mombasa**	Urban	7	2	9	36	0	36
**Total**		14	23	37	97	14	111

### Research team

Development of the qualitative study protocol and data collection instruments was overseen by an anthropologist trained in qualitative methodology [FAM] in partnership with the principal field investigator [NAO], a Kenyan expert in maternal and child health. Meetings between senior members of the Kenya-based data collection team and the US-based analysis team occurred on a weekly basis during the study to ensure the validity and cultural relevancy of all study materials, results, and conclusions. All data collection activities were performed by Kenyan team members who received training in interview protocol, note taking, consenting, data management, and transcription. One member of the US-based analysis team [SWN] was fluent in Swahili and was able to confirm local terms that remained in the translated interviews.

### Research instruments

An interview guide was developed using an iterative, team-based approach incorporating findings from the World Health Organization’s Strategic Working Group of Experts on Immunization, field notes from observations performed in antenatal clinics, and themes emerging from pregnant women interviews. The guide was revised after field testing with three interviews based on input from the Kenya-based data collection team. The final guide covered the following topics: (a) proportion of patients estimated to have received or refused maternal vaccines; (b) gestational age at which vaccines are given; (c) barriers or reasons for refusal cited by patients; (d) perceived ability and methods used to address these barriers/refusals; (e) comfort discussing vaccine recommendations with patients; (f) knowledge and experiences of vaccine preventable diseases; (g) existing patient education efforts; (h) introduction of future maternal vaccine recommendations into the current schedule; and (i) knowledge and attitudes surrounding tetanus toxoid (TT2+) and influenza vaccine effectiveness and safety.

### Data collection

Interviews were conducted in private rooms by teams of two members of the data collection team [WNW, SNW, ROA]. Written informed consent, covering interview participation, transfer of data to the US for analysis, and presentation of de-identified portions of interview transcripts, was obtained prior to the start of each interview. Face-to-face interviews were conducted in English or Swahili at participants’ places of employment, and in some cases, at their homes (to accommodate public sector workers who were on strike) between January and August 2017. Interviews were audio recorded, transcribed verbatim, translated where necessary by the data collection team, and assessed for accuracy by a team lead before analysis. No identifying information was included in written transcripts, which were stored on a secure server along with copies of the audio recordings. Original audio recordings were deleted immediately following transcription.

### Analysis

Principles of thematic analysis ^16^ informed our coding methodology. Codes were created both deductively and inductively through an iterative, team-based process based on interview guide questions and emergent themes captured by reading a sample of 12 transcripts from all sites. After finalization of the preliminary codebook, three transcripts representing different sites were selected and coded by the data analysis team [IB, SWN, CAA]. Inter-coder reliability was assessed using NVivo 11 Pro (QSR International Pty Ltd, Victoria, Australia), after which inconsistencies in coding were resolved and the codebook was revised to accommodate further emergent themes. After several iterations of this process, the codebook was finalized with inter-coder reliability at kappa >0.8 on 10% of transcripts representing all sites ( Supplementary File 2). All interview transcripts were coded using NVivo 11 Pro by a team of four research assistants [IB, SWN, CAA, VLF]. Thematic analysis of the coded transcripts was performed by the coding team [IB, SWN, CAA, VLF] and senior investigators [PMF, FAM].

## Data availability

Data underlying this study is available from figshare. Dataset 1: Healthcare Provider Interview Transcripts https://doi.org/10.6084/m9.figshare.6626498.v1

This data is available under a CC BY 4.0 license

## Supplementary material

Data collection and analysis instruments are available from figshare.

Supplementary File 1: Interview Guide: https://figshare.com/s/ae6477d762537b7e536a

Supplementary File 2: Codebook: https://figshare.com/s/be38088c7739b1462835

This data is available under a CC BY 4.0 license
