# Supplementary material for: Provider perspectives on demand creation for maternal vaccines in Kenya
Source: Gates Open Res. 2018 Jul 19;2:34. [Version 1] doi: 10.12688/gatesopenres.12833.1 (PMC6266652; doi:10.12688/gatesopenres.12833.1)
Supplement: Peer review history

# Peer review: Reviewer response for version 1

**DOI:** 10.21956/gatesopenres.13910.r26624

**Published:** 2018-10-18

**Authors:** Clare L. Cutland

**Affiliations:** 1 Faculty of Health Sciences, University of the Witwatersrand, Johannesburg, South Africa; 2 MRC Respiratory and Meningeal Pathogens Research Unit, Chris Hani Baragwanath Hospital, Johannesburg, South Africa; 3 DST NRF Vaccine Preventable Diseases, Chris Hani Baragwanath Hospital, Johannesburg, South Africa

This article presents the results of qualitative data collected during semi-structured interviews conducted as part of a large multi-component, mixed methods study, in four regions of Kenya. It aimed to describe issues around acceptance of maternal immunisation and demand creation by health care providers. The article is well written, with clear, concise descriptions of the six major themes which emerged from health care provider interviews. The findings concluded that HCP supported maternal immunisation, including the implementation of new vaccines, and that HCP play an important role in informing patients about availability and benefits of maternal immunisation. Additionally, ongoing training of HCPs and availability of educational resources for patients would be beneficial, as would regular engagement with religious and community leaders.

  This article is important and well timed, and contributes to the small, but expanding pool of data on perceptions about maternal immunisation.

 The participants were nurses and clinical officers who work in public and private health provider facilities in both rural and urban areas. Participant choice was good, as nurses and clinical officers have extensive contact with patients, and are able to educate and advise patients. The under-sampling of public health care providers is a limitation, which has been reported by authors.

I have read this submission. I believe that I have an appropriate level of expertise to confirm that it is of an acceptable scientific standard.

# Peer review: Reviewer response for version 1

**DOI:** 10.21956/gatesopenres.13910.r26575

**Published:** 2018-07-30

**Authors:** Paul A. Freeman

**Affiliations:** 1 Independent Consultant, Seattle, WA, USA; 2 Department of Global Health, University of Washington, Seattle, WA, USA

This paper is well presented with good quality methodology and analysis leading on to consistent conclusions. Semi-structured interviews of a convenience sample of facility based nurses and Clinical Officers  providing maternal and child health care were conducted according to a standardized interview. It was concluded that provision of standardized evidence-based print materials for patient education in-clinic may improve mothers' knowledge about maternal vaccines. Engagement of religious and community leaders, as well as male decision makers in the household were good recommendations to promote future acceptance of maternal vaccines. However, the discussion could be strengthened by referral to the evidence of the effectiveness of community-based approaches such as household visits by community health workers,women's groups and community outreach from health facilities in the provision of antenatal care and education about attendance for vaccines.( http://jogh.org/community-based-PHC.htm)

I have read this submission. I believe that I have an appropriate level of expertise to confirm that it is of an acceptable scientific standard.
